# Randomized, open-label, multicenter study of azithromycin compared with doxycycline for treating anorectal *Chlamydia trachomatis* infection concomitant to a vaginal infection (CHLAZIDOXY study)

**DOI:** 10.1097/MD.0000000000014572

**Published:** 2019-02-15

**Authors:** Olivia Peuchant, Edouard Lhomme, Marion Krêt, Bellabes Ghezzoul, Caroline Roussillon, Cécile Bébéar, Frédéric Perry, Bertille de Barbeyrac

**Affiliations:** a University Bordeaux; b INRA, USC EA 3671, Mycoplasmal and Chlamydial Infections in Humans; c CHU de Bordeaux, Bacteriology department, French National Reference Center for bacterial STI, Bordeaux, France; d CHU de Bordeaux, Pôle de santé publique, CIC1401-EC; e CHU de Bordeaux, Pharmacy Department; f CHU de Bordeaux, Safety and Vigilance Unit Department; g CHU de Bordeaux, Research and Clinical Study Department, Bordeaux, France.

**Keywords:** anorectal infection, azithromycin, *chlamydia trachomatis*, doxycycline, women

## Abstract

**Background:**

*Chlamydia trachomatis* can lead to a persistent infection in the lower gastrointestinal tract, suggesting a potential role of autoinoculation of cervical chlamydial infection from the rectal site, contributing to repeat infections. Moreover, around 75% of women with urogenital *C. trachomatis* have concurrent anorectal infection. Current treatment guidelines for urogenital *C. trachomatis* infection recommend either a single 1 g dose of azithromycin or doxycycline 100 mg twice daily for 7 days. Doxycycline appears to be more effective in treating anorectal infections as suggested in a population of men who have sex with men, but no randomized controlled trial (RCT) had directly compared azithromycin with doxycycline for the treatment of rectal infections. We propose an open-label RCT to compare the microbial cure obtained with a single 1 g dose of azithromycin versus 100 mg of doxycycline twice daily for 7 days, for the treatment of anorectal *C. trachomatis* infection concurrent to urogenital infection in women.

**Methods and study design:**

A total of 460 women with *C. trachomatis* urogenital infection will be enrolled in the study. Women will be asked to provide self-collected anorectal swabs and will be randomized to receive either a 1 g single dose of azithromycin or doxycycline 100 mg twice daily for 7 days. Clinical and biological data will be collected and patients will complete questionnaires about their sexual behavior. The primary outcome is the microbial cure rate, defined as a *C. trachomatis* negative nucleic acid amplification test (NAAT) result in the anorectal specimens 6 weeks after treatment initiation among women with a *C. trachomatis* positive urogenital and anorectal NAAT result at the baseline. The secondary outcome is autoinoculation from the rectum to the vagina, which will be evaluated based on the number of women with the same *C. trachomatis* genotype profile that will be identified in an anorectal-positive specimen obtained 6 weeks after treatment initiation and in a vaginal-positive specimen obtained four months after treatment.

**Discussion:**

The results of this trial will establish which treatment is more efficacious against anorectal infection and could affect recommendations for the treatment of urogenital *C. trachomatis* infection, taking into account concurrent anorectal infection.

**Trial registration numbers:**

EudraCT number: 2017-002595-15.

**ClinicalTrials.gov Identifier:**

NCT03532464. Date of registration: May 31, 2018.

**World Health Organisation International Clinical Trials Registry:**

NTC03532464. Secondary ID: CHUBX 2016/26. Date of registration: May 09, 2018.

## 1 Introduction

*Chlamydia trachomatis* is the most commonly reported bacterial sexually transmitted infection (STI). Screening studies have found large differences depending on the population being tested, ranging from 1% to 3% in the general population to 10% to 15% in individuals consulting an STI screening center, or among women requesting an abortion.^[1–4]^ Up to 75% of *C. trachomatis* infected women are asymptomatic. If not treated, these infections can lead to complications and sequelae such as pelvic inflammatory disease, ectopic pregnancy, and tubal factor infertility.^[5]^ In addition to infection of the urogenital tract, *C. trachomatis* can also cause anorectal infections, which are typically asymptomatic.^[6]^ The proportion of women having a rectal *C. trachomatis* infection among those positive for urogenital *C. trachomatis* ranges from 45% to 100%.^[7–11]^ Notably, there is no reported association with anal intercourse.^[7–11]^ Recently, it has been suggested that the anal transmission of *C. trachomatis* in women may occur by autoinoculation from the vagina due to the close proximity of the vagina and the anus.^[8,12,13]^ Moreover, it has been suggested that women become infected with *C. trachomatis* orally through various sexual activities and that the organisms could establish a persistent infection in the lower gastrointestinal tract where the immune response is downregulated, suggesting the potential role of autoinoculation of cervical chlamydial infection from the rectal site.^[14,15]^ Such repeated urogenital infections could lead to reproductive tract morbidity.

For uncomplicated *C. trachomatis* urogenital infections, recommended treatments are azithromycin 1 g orally as a single dose or doxycycline 100 mg orally twice daily for 7 days.^[16,17]^ Single doses are most likely to be adhered to, whereas following a week-long regimen of daily antibiotics is likely to make people feel sick and thus defer sexual activities.^[18,19]^ A meta-analysis of 23 randomized controlled trials (RCTs) comparing both treatments found an overall efficacy of 94.3% for azithromycin and 97.4% for doxycycline on urogenital infections.^[18]^

Adequate treatment for anorectal *C. trachomatis* is currently under debate. A recent meta-analysis found a pooled treatment efficacy of 82.9% for a single 1 g dose of azithromycin and 99.6% for 100 mg of doxycycline twice daily for 7 days.^[20]^ While this suggests that doxycycline may be a more effective treatment, it must be emphasized that the quality of the evidence was poor, as the meta-analysis was based entirely on observational studies, with 75% (6/8) of the studies being retrospective case note reviews. If rectal *C. trachomatis* is a hidden reservoir influencing transmission rates and considering the potential complications of cervical infections, providing further evidence of the need for effective rectal treatments among women is highly relevant. These circumstances highlight the need for RCT evidence comparing azithromycin with doxycycline for the treatment of anorectal *C. trachomatis* in women. Our hypothesis is that the efficacy of azithromycin for anorectal *C. trachomatis* infection may be lower than that of doxycycline, resulting in reinfection by autoinoculation from the rectum to the vagina.

## 2 Methods

### 2.1 Aim

The principal aim of this trial is to compare the microbial cure obtained with a single 1 g dose of azithromycin versus 100 mg of doxycycline twice daily for 7 days for the treatment of anorectal *C. trachomatis* infection concurrent to a vaginal infection in women, 6 weeks after treatment initiation.

### 2.2 Design

The Chlazidoxy trial is designed as a randomized, open-label, multicenter study comparing the efficacy (measured as the microbial cure) of a single 1 g dose of azithromycin versus 100 mg of doxycycline twice daily for 7 days for the treatment of concurrent anorectal and urogenital *C. trachomatis* infections. The trial will be conducted in accordance with the Declaration of Helsinki.

### 2.3 Setting

Women will be recruited in France in primary care from 4 free STI screening centers “CeGIDD” (“Centre Gratuit d’Information, de Dépistage et de Diagnostic”) and from 3 pregnancy termination centers after they had an abortion. We chose these centers because of the high prevalence of urogenital *C. trachomatis* infections (10*–*12%)^[3,4,21]^ and because the French National Authority for Health recommends screening for vaginal *C. trachomatis* infection in these populations.^[3,21]^

### 2.4 Patient characteristics

#### 2.4.1 Inclusion criteria

(1) Female
(2) Age ≥ 18 years
(3) *C. trachomatis* positive nucleic acid amplification test (NAAT) for vaginal specimen at baseline
(4) Women consulting STI screening centers with a negative β-hCG urinary assay and using efficacious contraception^[22]^
(5) Women requesting an abortion at a pregnancy termination center and with efficacious contraception after abortion
(6) Consultation in one of the participating centers
(7) Agree to follow-up contact
(8) Member or beneficiary of a social security system
(9) Written informed consent signed by the participant and the investigator (no later than the inclusion day and before performing any examination required for the study)

#### 2.4.2 Exclusion criteria

(1) Women with symptoms suggestive of pelvic inflammatory disease
(2) Prescribed an antibiotic with antichlamydial activity within 21 days before screening or between screening and enrolment
(3) Contraindications to tetracycline, macrolides, lincosamides, streptogramins, or ketolides (including allergy and treatment with colchicine, cisapride, ergot alkaloids, and retinoids)
(4) Inability to swallow pills
(5) Refusal to participate in the study
(6) Objectives of the study not understood
(7) Breastfeeding
(8) Women with serious cardiac diseases: QT interval prolongation, cardiac arrhythmia, advanced heart failure (NYHA > III)
(9) Women treated with anticoagulants or drugs with a definite potential of “*torsades de pointes”*
(10) Women with severe liver diseases

A person enrolled in the trial will not simultaneously participate in another one.

### 2.5 Context and procedure

#### 2.5.1 Recruitment

**Figure 1:**
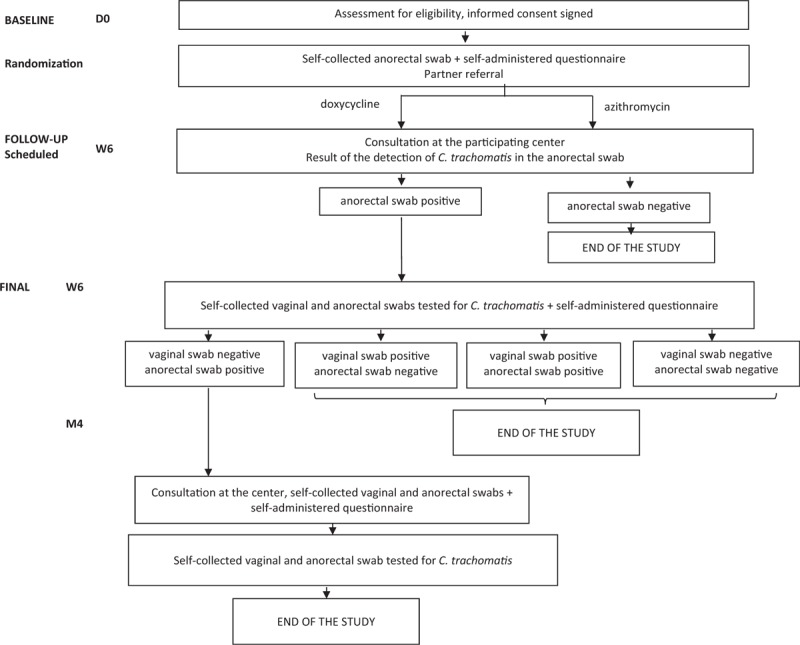
Design of the Chlazidoxy study.

Women with a *C. trachomatis* positive vaginal swab at the baseline will be approached by a research nurse and invited to take part in the trial (Fig. 1). The nurse will explain the trial and investigator will assess eligibility and obtain consent. Eligible women will be enrolled. Participants will provide a self-collected anorectal swab. Previous studies have found high feasibility and acceptability of self-collected anorectal testing in women who do not have an indication.^[10,11]^

All women with urogenital *C. trachomatis* infection will be included without waiting for the NAAT result of the anorectal specimen because treatment cannot be delayed. However, the study population will consist of only women with both *C. trachomatis* positive vaginal and anorectal swabs.

Information on the need for partner(s) to be treated will be provided. Sexual abstinence or protected intercourse with the partner(s) up to 7 days after the single dose of azithromycin or after 7 days of doxycycline will be recommended (usual recommendations).

The research nurse will complete an electronic Case Report Form (e-CRF) at the time of recruitment of the demographic characteristics (birth date, country of birth, marital status, education level, professional status), and biological (previous *C. trachomatis* infection, HIV-serology, *Neisseria gonorrhoeae* infection, hepatitis B virus, and syphilis serology) and clinical data (vaginal discharge, pelvic pain, abnormal bleeding, pain during sexual intercourse, anal discharge, anal itching, tenesmus). Participants will complete a questionnaire about their sexual behavior [regular sexual partner(s), occasional sexual partner(s) in the last 12 months, use of condoms, anal and oral intercourse] (Table 1).

**Table 1:**
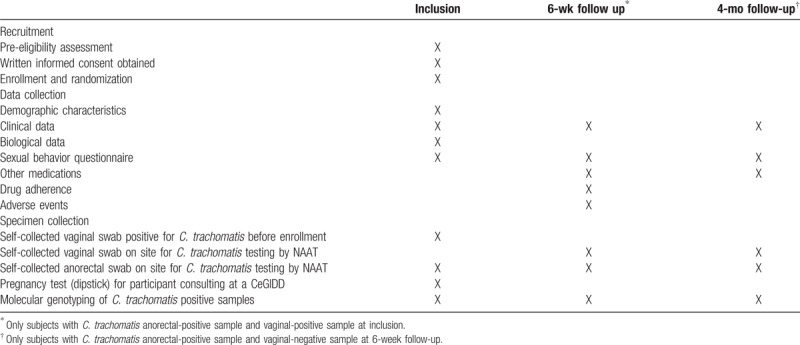
Trial timeline.

#### 2.5.2 Randomization

The randomized list will be generated centrally by an independent statistician before the start of the study and will be kept confidential in a secure environment. The sample sizes of each randomized group will be balanced with a 1:1 ratio. Randomization will be stratified in the investigating centers to take into account the potential differences in the characteristics of women consulting these centers. A validated web-based system will be used to implement the random assignment to each group.

After completion of the baseline assessment, the investigator will log onto the study e-CRF and complete the “Randomisation” web page with the eligibility criteria and validate the content. The website will then immediately list the treatment assigned to the trial participant.

#### 2.5.3 Intervention

Participants will be randomly allocated to one of the following treatment groups:

(1) Azithromycin: women in the azithromycin group will take four tablets of 250 mg as a single dose.
(2) Doxycycline: Treatment with doxycycline will consist of administration of 1 tablet of 100 mg of doxycycline twice daily in the morning and evening for 7 days. Doxycycline tablets will be taken at mealtimes with a glass of water (minimum 100 mL) and at least 1 hour before bedtime. Women will be advised to take antacids, iron supplements, zinc supplements, and didanosine at least 2 hours after taking the doxycycline. Women will be advised to minimize sun exposure or use sunscreen to reduce the risk of photosensitivity.

Antibiotics will be dispensed in their usual packaging with a clinical trial label.

#### 2.5.4 Follow-up at 6 weeks

All women will be asked to attend the participating center 6 weeks after treatment initiation.

During this visit, the research nurse will look at the *C. trachomatis* detection result of the anorectal swab performed at baseline. If no *C. trachomatis* was detected, the study will end. If the detection of *C. trachomatis* is positive, patient will be asked to provide new self-collected vaginal and anorectal swabs for *C. trachomatis* detection (Fig. 1).

The research nurse will complete the e-CRF questionnaire about drug adherence. Participants will return the treatment box for a pill count as a proxy measure of drug adherence. Women will be asked whether they have experienced any diarrhea or vomiting that could affect the levels of antibiotic absorption, or any adverse events. Clinical data during the last 6 weeks (vaginal discharge, pelvic pain, abnormal bleeding, pain during sexual intercourse, anal discharge, anal itching, and tenesmus) and information about whether they have taken any other antibiotics will be collected.

Participants will complete a questionnaire about their sexual behavior during the last 6 weeks [sexual intercourse with a regular or occasional partner, nature of the intercourse (genital and/or anal and/or oral), and use of condoms] and their partner's treatment (Table 1).

At this stage, the study will end for all women with *C. trachomatis* negative vaginal and anorectal swabs and for those with a *C. trachomatis* positive vaginal swab (whatever the result of the anorectal swab) (Fig. 1). Women with a *C. trachomatis* positive vaginal swab will be informed by the practitioner at each center, who will provide treatment and information on the need for their partner(s) to be treated (usual recommendations).

Women with a *C. trachomatis* negative vaginal swab and a *C. trachomatis* positive anorectal swab will be asked to attend the participating center 4 months after treatment initiation (Fig. 1).

#### 2.5.5 Follow-up at four months

Participants will provide self-collected vaginal and anorectal swabs for *C. trachomatis* detection.

The research nurse will collect information on the e-CRF about other antibiotics taken and clinical data since the last visit (vaginal discharge, pelvic pain, abnormal bleeding, pain during sexual intercourse, anal discharge, anal itching, and tenesmus) (Table 1).

Participants will complete a questionnaire about their sexual behavior since the last follow-up visit [sexual intercourse with a regular or occasional partner, nature of the intercourse (genital and/or anal and/or oral), and use of condoms] and partner's treatment.

Data collected regarding participants will be anonymized.

If the participant does not attend the follow-up visit scheduled, the study site coordinator of each center will contact her by phone to schedule a new appointment within the 2 following weeks. To compensate participants for the time dedicated to the study, financial compensation will be given at the follow-up visits.

In the case where participants do not complete follow-up, attempts will be made to obtain information on the reasons for dropout. The last contact will be recorded.

#### 2.5.6 Specimen processing

For each specimen, the detection of *C. trachomatis* will be carried with a routine NAAT at each laboratory of the corresponding participating center. All *C. trachomatis* positive specimens will be sent to the National Reference Centre for bacterial STI for genotyping (Table 1).

#### 2.5.7 Genotype determination

DNA will be extracted using the automated MagNA Pure 96 isolation and purification systems (Roche Diagnostics, Meylan, France). A method based on sequencing of the *omp1* gene will be performed to determine the strain genovar on sequential samples.^[23]^ If the genovars are the same, a molecular method based on multilocus variable number tandem repeat analysis (MLVA),^[24]^ which is able to discriminate strains belonging to the same genovar, will be performed. Both methods can be directly applied to the clinical specimens without a cell-culturing step.

### 2.6 Outcomes

#### 2.6.1 Primary outcome

The primary outcome is the microbial cure rate defined as a C. *trachomatis* negative NAAT result in anorectal specimens 6 weeks after treatment initiation (with azithromycin and doxycycline) among women with a C. *trachomatis* positive vaginal and anorectal NAAT result at the baseline.

#### 2.6.2 Secondary outcomes

We will estimate the prevalence of anorectal *C. trachomatis* infection concurrent with urogenital infection. The prevalence will be defined at the baseline by the number of women with an anorectal *C. trachomatis* infection divided by the total number of women included in the study.

For each participant, we will further determine the *C. trachomatis* genotype profile in order to identify whether the same *C. trachomatis* genotype profile is found in the vaginal-positive and anorectal-positive specimens.

### 2.7 Sample size

#### 2.7.1 Pilot study

We conducted a pilot study to evaluate the percentage of women with concurrent urogenital and anorectal *C. trachomatis* infections, in the same population and at the same centers as the trial, to measure the size of the population to be tested. A total of 100 women with a self-collected vaginal swab positive for *C. trachomatis* using NAAT were included and provided self-collected anorectal swabs for *C. trachomatis* detection. Overall, 77.1% (74/96) were positive for anorectal *C. trachomatis*. These results highlight the feasibility of our study.

#### 2.7.2 Sample size calculation

We assume rates for successful treatment of anorectal *C. trachomatis* infection of 99% for doxycycline and 83% for azithromycin and a loss of follow-up of 10%. Missing values will be replaced by a failure of the treatment in the intention-to-treat primary analysis. Therefore, to compare 89.1% versus 74.7%, with a 2-sided type 1 error rate of 5% and a power of 90% for a Chi-squared test, the sample size is estimated at 149 patients per group (SAS, proc power v9.3).

According to the pilot study, the prevalence of anorectal *C. trachomatis* infection concurrent with urogenital infection was 75% with 95% confidence interval (95% CI) = (65*–*84%) in our population. For our trial, we made the conservative assumption of a prevalence of 65% of anorectal *C. trachomatis* infection concurrent with urogenital infection at inclusion; therefore, we will include 230 women per group and 460 women in total.

With a total of 460 women enrolled, we will be able to estimate the prevalence of concurrent anorectal and urogenital infections with the following precision:

(1) for a prevalence of 65%: 95% CI = (60.5*–*69.3)
(2) for a prevalence of 75%: 95% CI = (70.8*–*78.9)

According to the data of the 7 participating centers in 2015, we have a potential number of 1115 eligible women with *C. trachomatis* positive vaginal specimens.

### 2.8 Analysis

Analyses will initially be conducted according to the intent-to-treat principle, with a “missing = failure” strategy for handling missing data on the primary endpoints, in which all randomized participants will be included in the group in which they were first randomized and all their data will be used, regardless of changes over the study duration. For the primary outcome, the failure will be defined as a *C. trachomatis* positive NAAT result for an anorectal swab at week 6, which indicates failure of the treatment.

To check the robustness of the main analysis to missing data, a sensitivity analysis will be performed:

(1) The maximum bias strategy: missing data will be replaced by a failure of the treatment in one group and by a treatment success in the other, and vice versa. Success is defined as a *C. trachomatis* negative NAAT result for an anorectal swab at week 6.
(2) An “under treatment analysis” will be performed in which participants will be included in the group according to the treatment they really received, with the available primary endpoints.

If the distribution of baseline prognostic factors is unbalanced between the groups despite randomization, the main and sensitivity comparative analyses will be performed with adjustments to those factors.

For each variable, the number of missing data will be described and the reason for any missing data will be documented as extensively as possible.

All the statistical tests will be performed with a 2-sided type I error rate of 5% and all CIs will be bilateral with a type I error rate of 5%.

Qualitative variables will be described in terms of number, proportion, and exact binomial CI of proportion. Quantitative variables will be described in terms of absolute frequency, mean, standard deviation, CI of the mean, median, minimum, maximum, 1st, and 3rd quartiles.

Descriptive analysis will be overall and by treatment group. Graphical analyses will be associated when possible.

The analyses will be performed using SAS software (version 9.3 or later; SAS Institute, Inc., Cary, NC).

### 2.9 Ethics approval

The Chlazidoxy trial has been approved by the French ethic Committee for the Protection of Persons (“Comité de Protection des Personnes”) (CPP) on February 14, 2018 (CPP number 2017-74-2). This trial was authorised by the French National Agency for Medicines and Health Products Safety (“Agence Nationale de Sécurité du Médicament et des Produits de Santé”) (ANSM) on January 26, 2018 (reference IDRCB 2017-002595-15). All participants in the study will sign an informed consent form before participation.

In case of a modification to the protocol, the investigator-coordinator is expected to submit a request to the CPP and send an information note to all the investigators.

### 2.10 Trial governance

#### 2.10.1 Sponsor

The Chlazidoxy trial is sponsored by the Bordeaux University Hospital.

Bordeaux University Hospital is involved in the implementation of the trial, legal/ethical submissions, and hosting of the Chlazidoxy database. It is not involved in the design of the study, analysis, or interpretation of the data. Bordeaux University Hospital is also in charge of regular quality control assessments. A clinical research assistant will visit participating centers to ensure that implementation is in accordance with the protocol.

Bordeaux University Hospital has taken out insurance from HDI-GERLING through BIOMEDIC-INSURE for the full research period, covering its own civil liability and that of any agent (doctor or research staff), in accordance with Article L.1121-10 of the French Public Health Code.

#### 2.10.2 Coordinating unit

Bordeaux University Hospital and the Bacteriology department are responsible for coordinating this trial. They will ensure that recruitment and follow-up are performed as planned.

#### 2.10.3 Investigating centers

CeGIDD and pregnancy termination centers are involved in the recruitment, inclusion, and follow-up of the participants. They collect informed consent from the participants and implement randomization. They are responsible for notify the sponsor's vigilance division of any adverse event by filling in the adverse event section of the e-CRF.

#### 2.10.4 Steering committee

The steering committee comprises coordinating investigators, scientific experts, a methodologist and biostatistician, and representative of the sponsor. The Scientific Committee of the study meets before the beginning of the inclusions, at the end of the inclusions, at the end of the study, and according to the needs defined by the promotor and the coordinating investigator. The steering committee will be responsible for inquiring about research progress, monitoring compliance with good clinical practice and patient safety, deciding on any relevant modification of the protocol, and monitoring compliance with the rules of communication and publication of the results described in the protocol.

#### 2.10.5 Data monitoring committee

The present study does not require an independent data monitoring board, given that both investigational drugs are already recommended at the same dosage by the French Authority for Public Health as the treatment of choice for chlamydia infections.

## 3 Discussion

Given the high prevalence of anorectal *C. trachomatis* infection concurrent with urogenital infection, it is important that the most efficacious treatment is used. Currently, as anorectal screening is not widely recommended in women with urogenital infection, azithromycin, which fails in 17% of cases according to meta-analysis, continues to be largely used. These findings suggest that many anorectal infections are suboptimally treated with azithromycin, which could be a potential obstacle in the control of *C. trachomatis* transmission and its complications. This will be the first RCT to compare azithromycin and doxycycline for concurrent anorectal and urogenital *C. trachomatis* infections in women and will establish whether doxycycline is more efficacious than azithromycin. If azithromycin efficacy is indeed 83%, then this is much lower than the 95% threshold recommended by the World Health Organization for STI treatment and it should not be used for rectal *C. trachomatis.*^[25]^ The ability to differentiate between treatment failure and reinfection using highly discriminative molecular methods is a major strength of this study.

We originally planned to realize this RCT as double-blind to ensure that the risk of reinfection was similar between treatment arms; it is possible that taking a daily dose (as is required for doxycycline) may deter people from resuming sexual activity while undergoing treatment.^[26]^ However, because of drug manufacturing reasons, we will have to conduct this study as an open-label RCT. Indeed, the treatments administered are those recommended by the French National Authority for Health and are very well accepted by all the patients; in addition, patients will be randomized with a 1:1 ratio. Both participants and investigators will have knowledge of the treatment arms. Despite the absence of blinding, the biologist performing the bacteriological analyses on the primary outcome will be blinded to the drugs taken by the participants, thus limiting the possibility of bias.

If our study reveals that doxycycline is more effective than azithromycin in treating anorectal *C. trachomatis* infections, the current recommendations for the treatment of urogenital *C. trachomatis* infections could be revised, taking into account a concurrent anorectal infection. The results of this study could challenge the consensus treatment consisting of a single 1 g dose of azithromycin in the case of uncomplicated urogenital infections. This may be restricted to use in urogenital infections without concurrent anorectal infection.

If our study is conclusive, an alternative strategy for *C. trachomatis* control would be to curtail the use of azithromycin. Moreover, azithromycin has the disadvantage of promoting resistance to macrolides of *N. gonorrhoeae* and *Mycoplasma genitalium*, the bacteria responsible for STIs that may be associated with *C. trachomatis*. Finally, the ultimate recommendation could be to treat *C. trachomatis* only with doxycycline, as was proposed for the control of gonorrhea and chlamydia in the United Kingdom in the past^[27]^ and as recommended in the European guidelines on *M. genitalium* infections published in 2016.^[28]^

## Acknowledgments

In addition to the Authors of this paper, the following people are Members of the Chlazidoxy study group: Dr Dounia Baïta, Gynecology department, Bordeaux University Hospital

Dr Pervenche Martinet, CeGIDD, Marseille, France

Dr Anne Grob, Biology department, Marseille, France

Dr Isabelle Le Hen, CeGIDD, Bordeaux, France

Dr Sophie Zaffreya, Medical Biology laboratory, Bordeaux, France

Dr Claire Bernier, CeGIDD, Nantes, France

Dr Sophie Anne Gibaud, Bacteriology department, Nantes University Hospital, Nantes, France

Dr Nathalie Trignol-Viguier, Gynecology department, Tours University Hospital, Tours, France

Dr Philippe Lanotte, Bacteriology department, Tours University Hospital, Tours, France

Dr Philippe Lefebvre, Gynecology department, Roubaix University Hospital, Roubaix, France

Dr Anne Vachée, Bacteriology department, Roubaix University Hospital, Roubaix, France

Dr Thomas Girard, Unité Guy Moquet, Espace Santé Jeune, Hôpital Hôtel Dieu, Paris, France

Dr Julien Loubinoux, Bacteriology department, Hôpital Hôtel Dieu, Paris, France

Christelle Turuban, Research and Clinical study department, Bordeaux University Hospital, Bordeaux, France

Chlazidoxy study group: Dounia Baïta, Pervenche Martinet, Anne Grob, Isabelle Le Hen, Sophie Zaffreya, Claire Bernier, Sophie Anne Gibaud, Nathalie Trignol-Viguier, Philippe Lanotte, Philippe Lefebvre, Anne Vachée, Thomas Girard, Julien Loubinoux, Christelle Turuban.

The authors report no conflicts of interest.

**Trial status**

The first enrolment date was October 19, 2018. The estimated last enrolment date will be in October 2019. The estimated follow-up completion date will be in January 2020.

**Additional files**

Additional file 1: Standard Protocol Items: Recommendations fir Interventional Trials (SPIRIT) 2013 checklist: recommended items to address in a clinical trial protocol and related documents.

**Availability of data and materials**

All personal and identifying information collected from participants will be kept in a secure place at each recruiting center during the duration of the trial and will be destroyed at the end of the study. Personal information will not be disclosed outside of the recruiting centers. The final dataset will be accessible only by the sponsor and the investigator-coordinator's unit.

## Author contributions

OP and BdB were major contributors to the conception of the protocol. EL, CR, BG, MK, and CB were involved in the conception of the protocol. OP, BdB, and EL were major contributors to the writing of the protocol. All authors are involved in the implementation of the trial. All authors have read and approved the final manuscript.

**Conceptualization:** Olivia Peuchant, Edouard Lhomme, Bertille de Barbeyrac.

**Data curation:** Edouard Lhomme, Marion Krêt.

**Formal analysis:** Olivia Peuchant, Edouard Lhomme, Marion Krêt, Bertille de Barbeyrac.

**Funding acquisition:** Olivia Peuchant, Frédéric Perry, Bertille de Barbeyrac.

**Investigation:** Olivia Peuchant, Bertille de Barbeyrac.

**Methodology:** Olivia Peuchant, Edouard Lhomme, Marion Krêt, Frédéric Perry, Bertille de Barbeyrac.

**Project administration:** Olivia Peuchant, Frédéric Perry, Bertille de Barbeyrac.

**Resources:** Bellabes Ghezzoul.

**Software:** Edouard Lhomme, Marion Krêt.

**Supervision:** Olivia Peuchant, Edouard Lhomme, Frédéric Perry, Bertille de Barbeyrac.

**Validation:** Olivia Peuchant, Edouard Lhomme, Bellabes Ghezzoul, Caroline Roussillon, Frédéric Perry, Bertille de Barbeyrac.

**Visualization:** Olivia Peuchant, Frédéric Perry, Bertille de Barbeyrac.

**Writing – original draft:** Olivia Peuchant, Edouard Lhomme, Bertille de Barbeyrac.

**Writing – review & editing:** Olivia Peuchant, Edouard Lhomme, Marion Krêt, Bellabes Ghezzoul, Caroline Roussillon, Cécile Bébéar, Frédéric Perry, Bertille de Barbeyrac.
